# On the Detection of Spectral Emissions of Iron Oxides in Combustion Experiments of Pyrite Concentrates

**DOI:** 10.3390/s20051284

**Published:** 2020-02-27

**Authors:** Carlos Toro, Sergio Torres, Víctor Parra, Rodrigo Fuentes, Rosario Castillo, Walter Díaz, Gonzalo Reyes, Eduardo Balladares, Roberto Parra

**Affiliations:** 1 Dirección de Investigación, Universidad Tecnológica de Chile INACAP, Avenida el Condor 720, Ciudad Empresarial, Huechuraba, Santiago RM858000, Chile; 2 Metallurgical Engineering Department, University of Concepción, Concepción CCP4070386, Chile; vparras@udec.cl (V.P.); walterdiaz@udec.cl (W.D.); gonzaloreyes@udec.cl (G.R.); eballada@udec.cl (E.B.); rparra@udec.cl (R.P.); 3 Electrical Engineering Department, University of Concepción, Concepción CCP4070386, Chile; sertorre@udec.cl; 4 Department of Analytical and Inorganic Chemistry, Faculty of Chemical Sciences, University of Concepción, Concepción CCP4070386, Chile; rodrigoal@udec.cl; 5 Department of Instrumental Analysis, Faculty of Pharmacy & Biotechnology Center, University of Concepción, Concepción CCP4070386, Chile; rosariocastillo@udec.cl

**Keywords:** optical sensors, combustion, spectral measurements, signal detection, signal reconstruction, signal processing, principal component analysis, multivariate data analysis

## Abstract

In this paper, we report on the spectral detection of wustite, Fe(II) oxide (FeO), and magnetite, Fe(II, III) oxide (Fe_3_O_4_), molecular emissions during the combustion of pyrite (FeS_2_), in a laboratory-scale furnace operating at high temperatures. These species are typically generated by reactions occurring during the combustion (oxidation) of this iron sulfide mineral. Two detection schemes are addressed: the first consisting of measurements with a built-in developed spectrometer with a high sensitivity and a high spectral resolution. The second one consisting of spectra measured with a low spectral resolution and a low sensitivity commercial spectrometer, but enhanced and analyzed with post signal processing and multivariate data analysis such as principal component analysis (PCA) and a multivariate curve resolution—the alternating least squares method (MCR-ALS). A non-linear model is also proposed to reconstruct spectral signals measured during pyrite combustion. Different combustion conditions were studied to evaluate the capacity of the detection schemes to follow the spectral emissions of iron oxides. The results show a direct correlation between FeO and Fe_3_O_4_ spectral features intensity, and non-linear relations with key combustion variables such as flame temperature, and the combusted sulfide mineral particle size.

## 1. Introduction

The copper pyrometallurgy industry is facing many challenges regarding the increase of production and more strict demands on environmental compliance regulations. In this context, the need for developing online sensing technologies aimed at monitoring the temperature and the chemical composition of the molten phases during the slag and copper tapping period and during the copper concentrate feeding is crucial to enhance the energy and production efficiency, gas emission control and general optimization of the copper making processes [1,2]. Thus, active and passive spectroscopy techniques have been tested to determine on-line relevant process information to achieve the combustion goals.

The most important smelting technology for copper concentrates is the flash smelting process where solid and fine particles of copper concentrate, composed mainly of chalcopyrite (CuFeS_2_), pyrite (FeS_2_) and bornite (Cu_5_FeS_4_), are fed to the smelter with oxygen-enriched air that promotes the combustion (oxidation). The combustion inside the reactor form two molten phases in the settler located at the bottom, namely, the matte that contains most of the copper fed as sulfide, and the slag having most of the iron fed as oxide. Since its implementation in the last century this has been continuously investigated in order to elucidate phenomena occurring in the reaction shaft that are not yet well understood.

Upon the ignition, the copper concentrate particles are instantaneously oxidized and a reacting flame is formed, which is promoted by the heat released by the exothermic oxidation reactions and the high temperature of the reaction shaft. The oxidation process of the concentrate particles should be conducted under conditions that further the formation of wustite (FeO) and prevent the excessive production of magnetite (Fe_3_O_4_) and cuprite (Cu_2_O). For the industrial method this can be related to a favorable distribution of oxygen in the reaction shaft and therefore a good performance of the smelting process is achieved. When the flash smelting reactor is operated under these conditions, the slag cleaning process is improved by handling low-magnetite and fluid slags, and consequently, decreasing copper losses [3]. It should be noted that with a FeO and Fe_3_O_4_ detection system it will be possible to prevent the over-oxidation of iron by adjusting the oxygen supply and other operational parameters based on the flame monitoring information.

As reported in the literature [2,4,5,6], spectroscopy and optical techniques are suitable, nondestructive and contactless sensing alternatives to characterize combustion processes. In particular, some authors have reported the FeO spectral emission pattern in the visible (VIS) spectral range as reported by West and Broida in their work [7]. They reported this emission as a continuum spectrum from 500–700 nm containing three main spectral features around 570 nm, 590 nm (which may interfere with sodium emission) and 620 nm. Furthermore, some authors reported FeO emission in meteoric ablation measurements and a terrestrial night airglow spectrum [8,9] as well as in experiments with induced emission on steel surfaces [10]. On the other hand, other authors reported spectral features at 605 nm and 615 nm as copper oxide (CuO) [11,12], and recently, Knapp et al. [13] proposed a model of this diatomic molecule. We were unable to find reports related with Fe_3_O_4_ spectral emissions in the VIS-NIR spectral regions for combustion processes.

To the best of our knowledge, there are no reported online industrial monitoring systems aimed at the detection of the presence of iron oxides or any other species in the copper flash smelting process. Only results obtained during laboratory scale experiments and exploratory industrial measurements have been reported to date in this field. Arias et al. [2] reported some spectral features found in radiation emitted from copper concentrate flash smelting and applied optical pyrometry techniques for temperature estimation. Diaz et al. [6] applied multivariate data analysis methods to describe and to classify spectral information from the combustion of pure copper and iron sulfide minerals. The present work aims to provide new insights on the spectral characterization of such processes, and it is specifically focused on the detection of weak FeO and Fe_3_O_4_ spectral emission signals.

The main goal of this manuscript is to apply spectral measurement techniques and mathematical methods to analyze the combusted iron sulfide pyrite spectral information to detect molecular emission intensities. The first procedure involves measurements with a high spectral resolution custom-made spectrometer (0.03 nm) and signal conditioning methods. The second procedure involves calibrated measurements with a commercial medium spectral resolution (0.12 nm) spectrometer, signal processing techniques and multivariate methods such as the principal component analysis (PCA) [14], for an exploratory analysis. The multivariate curve resolution—alternating least squares (MCR-ALS) algorithm is employed to separate the spectral signals into pure components and relative concentration profiles [15]. This method has been mainly applied in the research field of chemometrics, to analyze, for example, hyperspectral images [16]. Finally, a non-linear model based on physical laws and the estimated spectral features with the MCR-ALS method is proposed to reconstruct the spectral signal produced during pyrite combustion. The paper is organized as follows: in Section 2, the test bench, and the experimental methodologies, as well as practical issues regarding the spectral measurements, are fully depicted. In addition, some radiometric issues regarding the process of interest are also discussed, and some theoretical aspects concerning the signal processing techniques applied to the measured spectra are reviewed. In Section 3, the main results of this research, and a discussion related to develop real time FeO and Fe_3_O_4_ monitoring systems, are presented. Finally, some concluding remarks and future work are outlined.

## 2. Materials and Methods

The experimental setup, the combustion tests carried out to detect the iron and copper oxides spectral features of interest, and our built-in high spectral resolution spectrometer are described in this section. First, note that whenever a spectrum from a flame is measured, its spectral behavior can be modeled as *I*(*λ*,*T*) = *I_c_*(*λ*,*T*) + *I_d_*(*λ*,*T*) + *I_mol_*(*λ*,*T*) + *e + d*, where *λ* is the sampling wavelength in nm, *T* is the temperature associated to the emission spectra, **I***_c_* is a continuous component or baseline spectrum, **I***_d_* and **I***_mol_* are excited elemental/discontinuous and molecular emissions respectively, *e* is a normal distributed electronic noise and *d* is an offset associated to spectrometer dark current noise [17]. The number of sampling wavelengths and spectral range are limited by the spectrometer detector itself. The implemented methods are described below.

### 2.1. Experimental Setup and Samples Preparation

**Figure 1:**
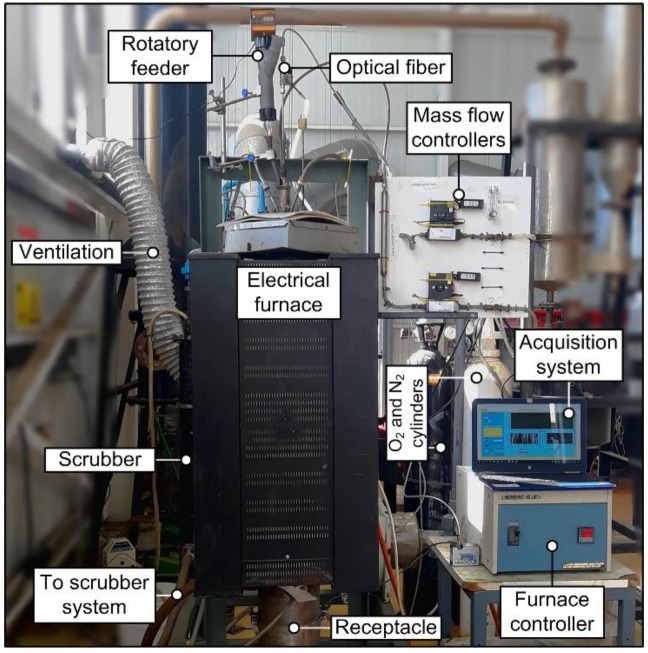
Experimental setup [6].

The pyrite combustion experiments are performed in a drop-tube arrangement, consisting of a cylindrical stainless-steel reaction zone, heated by a controlled electrical furnace. The laboratory scale combustion setup highlighting the main components can be seen in Figure 1. The furnace operating temperature is set to 1273 K for all tests. Combustion of sulfide particles was promoted with oxygen enriched mixtures containing N_2_. Oxygen and nitrogen flow rates were adjusted to have a laminar flow regimen inside the reaction zone.

In this study high-grade pyrite samples, provided by Ward’s Science (Rochester, NY, USA), were used as raw materials during the combustion experiments. The pyrite was dry-sieved and separated into six target size fractions: <37 µm, 37 to 44 µm, 44 to 53 µm, 53 to 74 µm, 74 to 105 µm and 105 to 149 µm. Prior to the experiments, all the sieved fractions were dried in a laboratory oven at 378 K for 24 h [18]. The dried samples were further labeled and stored in sealed plastic bags. The particle size distribution of all the raw materials and the oxidation products was determined by means of a Sympatec Helos-Sucell^TM^ particle size analyzer (Sympatec GmbH, Clausthal-Zellerfeld, Germany) based on a laser diffraction technique. Automated mineralogical characterization of minerals and the combustion products was determined using a Quantitative Evaluation of Minerals by SCANnig electron microscopy (QEMSCAN^®^) system, produced by the Fei Company (Hillsboro, OR, USA). The amount of magnetite in the oxidized products was determined by means of a Satmagan balance and used to quantify the magnetite to hematite ratio from the results obtained by means of QEMSCAN^®^ analysis.

### 2.2. Spectral Acquisition System

The optical sensing system consisted of a specially designed multicore VIS-NIR optical fiber (O.F.) (Avantes Ltd., Apeldoorn, The Netherlands) of ~2 m, with its own cooling system to perform measurements in high temperature environments. Special care was taken to avoid any dust deposit in the optical fiber tip, to achieve this: we ensured a laminar flow regimen inside the drop-tube; we used the same cooling nitrogen flowing through the optical fiber tip, at a flow rate of ~2 L/min, as a dust blower system and; we ensured a negative pressure through the exhaust gas system with an extractor fan.

The O.F. cable, composed by an arrangement of seven individual O.Fs, was used just as a light guide in this work. The O.Fs. transmit the combustion radiation to a USB4000 spectrometer, operating in the spectral range from 344.26 nm to 1034.34 nm. Calibration is performed with a HL-2000-CAL lamp to measure the combustion intensity radiation in absolute irradiance units of µW/[nm∙cm^2^] (both manufactured by Ocean Optics Inc., Dunedin, FL, USA). In this work, the effective transduced spectral range is trimmed to 400–900 nm due to silicon detector signal to noise ratio issues, leading to *n* = 2576 spectral samples. Concerning the O.F.-spectrophotometer system a calibration procedure and two references were needed: (i) a dark noise reference *d*, measured by blocking the optical fiber tip, and (ii) a standard calibration source reference with a broad band radiance signature. Then, since the true calibration lamp radiance signature is provided by the manufacturer and the system transfer function *H*(*λ*) in units of µJ/counts was estimated, a calibration for a new raw measurement **I***_raw_* can be performed, which can be obtained by a calibrated spectrum, **I***_cal_* as follows:

(1) $$I_{cal}(\lambda_{i})=\frac{\left(I_{raw}(\lambda_{i})-d_{i}\right)[counts]\cdot H(\lambda_{i})[\mu J/counts]}{t_{\mathrm{int}}[\mathrm{seg}]\cdot A_{c}[{\mathrm{cm}}^{2}]\cdot \Delta \lambda_{i}[\mathrm{nm}]}$$

where *d_i_* is the dark reference at pixel *i*, *i* = 1, …, *n*, *t*_int_ is the integration time, *A_c_* is the system collection area, assumed as a single O.F. cross section connected to the spectrometer and equal to π∙(0.5∙D_OF_)^2^, with D_OF_ as its core diameter, which is 400 µm for our system. In addition, Δ*λ_i_* indicates the band of nanometers represented by each pixel *i* inside the spectrometer linear CCD array, which can be estimated as Δ*λ_i_* ≈ (*λ_i_*_+1_ − *λ_i_*_−1_)/2 ≈ (*λ_i_*_+1_ − *λ_i_*). Although this is a straightforward approach, numerical errors can appear caused by the discrete differences. Therefore, it is recommended to model the provided wavelength vector as a second order polynomial *λ_i_* = p(*i*) first and then to calculate the analytical derivative *dλ_i_* in order to estimate Δ*λ_i_* for (1).

**Figure 2:**
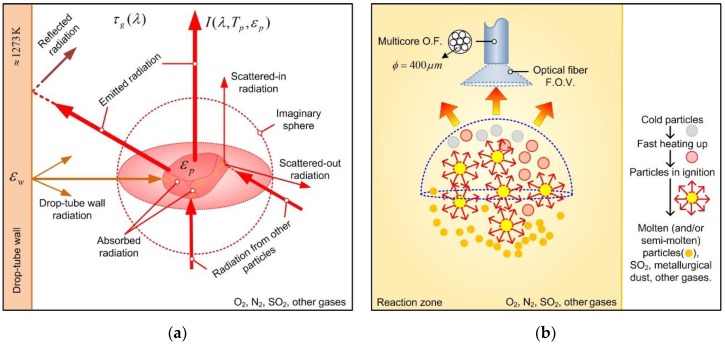
Radiometric measurement scheme and associated radiative processes: (**a**) Single heated particle radiative emission with its surroundings; (**b**) sensing scheme depicting the different particle states during their passing through the reaction zone.

To measure the combustion flame radiation is a complex task, involving the following chemical and physical processes: radiation emissions from gas-solid-liquid phases, radiation coming after the process of absorption, scattering and; surrounding drop-tube wall emissions, absorptions, and reflections. A scheme showing the involved radiative process is depicted in Figure 2a.

In Figure 2b, we illustrate a simple combustion scheme for sulfide particles covering some physical phase changes that a particle can experience inside the reaction zone. For a detailed discussion regarding these reactions please refer to [18]. In this figure, the O.F. field of view (F.O.V.) was assessed by connecting the O.F. to a standard halogen lamp and then by measuring the illuminated circular surface produced at a fixed distance, leading to a value of ≈26°. Although, the reported F.O.V. is wide enough to capture radiation from the flame and from the drop-tube wall, we neglected the last source since it could be visually confirmed that the main contribution is caused by the combustion flame radiation. We include in the results section an analysis of background radiation to discard any parasite molecular or atomic emission that could reach the O.F. tip. In order to simplify the spectral analysis, three main assumptions were considered in this work:

(i) The gases surrounding the combustion flame are mainly SO_2_, N_2_ and O_2_. They, at experimental temperatures ≈ 1000 K to 2500 K have a transmittance τ*_g_*(*λ*) = 100% in the 400 nm to 900 nm spectral band.
(ii) It is assumed that combustion flame emits as a gray body, i.e., it presents a constant spectral emissivity behavior. A discussion is given at the end of this work regarding this assumption.
(iii) The main contribution to detected radiation is due to molten, in-ignition and heated particles during the combustion experiments. Drop-tube wall emissions and wall reflected radiation are neglected in this work.

Spectral signal acquisition and spectrometer configurations were performed with an on purpose virtual instrument developed in LabView^TM^ software (National Instruments Corporation, Austin, TX, USA), and all computations and multivariate data analysis were performed with Matlab^TM^ software (The Mathworks, Inc., Natick, MA, USA).

### 2.3. Experimental Design

In this work, 6 sets of combustion experiments were conducted to sense the iron oxide spectral features. They consisted of combustion experiments of a high-grade pyrite samples with 6 different particle sizes. These sets are: 105 to 149, 74 to 105, 53 to 74, 44 to 53, 37 to 44 and <37 µm. From now on the samples are labeled as PyA, PyB, PyC, PyD, PyE and PyF respectively to the previous order. The particle size distribution was assessed with laser diffraction techniques. The gas supplied in each experiment consisted of an oxygen/nitrogen mixture, in which the oxygen concentration was set to 80 percent by volume. In addition, the oxygen concentration in the mixture was set to supply three times the stoichiometric amount to completely oxidize the sulfide species in the samples to form SO_2_ and Fe_3_O_4_, according to the reaction: FeS_2_(s) + 8/3O_2_(g) → 1/3Fe_3_O_4_(s) + 2SO_2_(g).

### 2.4. High Spectral Resolution Spectrometer Development

In order to measure the FeO spectral emitted signature from the pyrite experiments and to resolve a particular atomic emission around 589 nm that was reported in [2], a high spectral resolution spectrometer was designed and developed. This particular atomic emission is known for showing up in spectral combustion measurements, and it is roughly assumed to behave as sodium (Na). The developed device operates with the well-known Ebert Fastie optical configuration with a 200 mm focal length at F/8 aperture, with a diffraction grating of 1200 lines/mm. Two spectral ranges were set, a VIS range and a NIR range. In its current stage of development, it presents low rate acquisition times when adjusted to detect weak emission signals. In Table 1 the main characteristics of the developed spectrometer are summarized.

### 2.5. Flame Spectral Data Signal Processing

In this work, signal processing techniques and multivariate data analysis were applied to analyze, and to extract meaningful features from collected spectra. Among them: (i) PCA for an exploratory analysis, (ii) MCR-ALS to disintegrate original spectra into reliable pure spectral components and, (iii) a non-linear least squares curve fitting procedure method, based on Planck’s radiation law, to estimate the combustion temperature.

PCA is used as a first approximation to discover weak spectral emission patterns hidden by noise. This multivariate analysis technique is mainly used for dimensionality reduction of multivariable data sets. Consider a set of *m* measured spectra composed, each sampled at *n* wavelengths and then spectra can be organized in a data matrix **X***_m_*_×*n*_ with each spectrum given by ***x****_s_* = (*x_s_*_1_ … *x_sn_*), *s* = 1, …, *m*. By means of PCA, *k* principal directions ***v****_k_*, orthonormal axes, can be extracted, in which the retained data variance is maximal [14]. In this work, these principal directions ***v****_k_* were calculated with the singular value decomposition algorithm over the centered data matrix, where each variable, *x_si_*, *i* = 1, …, *n*, is centered by subtracting the respective column mean ${\bar{x}}_{i}$ according to $x_{si}-{\bar{x}}_{i}$, as shown in Figure 1a.

Each of the principal directions, named as loadings or principal vectors (PV) as well, allows weak spectral patterns to emerge from electronic noise and the continuum high intensity spectral radiation. Although the amplitude of a PV does not have physical or chemical quantitative meaning, the structure of such signals uncovers molecular and elemental emissions encrypted in the original data.

To perform a reliable separation of the pure spectral signals composing a spectrum, the MCR-ALS algorithm was applied over the spectral data with highest emission profiles. This method assumes that an experimental data matrix **D** can be modeled as **D** = **CS**^T^ + **E**, where **C** and **S**^T^ contain pure profiles related to relative concentrations and spectra respectively [15] and **E** is the matrix of residuals not explained by the resolved components. The MCR-ALS algorithm was implemented with a Matlab^TM^ graphical user interface (GUI) provided by Tauler and collaborators. Their GUI, code and tutorials are available at the website: http://www.mcrals.info. Let us recall that one of the aims is to determine the main *k* molecular and line emission spectral components present in the measured data. Then, the obtained spectra are used in a curve fitting procedure to analyze each spectrum separately. To perform a complete estimation of the *k* + 3 involved parameters: the combustion temperature, *T_s_*, the relative concentrations of pure spectral features, **I***_k_*, composing a measured spectrum **I***_s_*, and a bias coefficient, *θ_s_*, accounting for non-corrected offsets during the calibration process. Thus, the proposed measuring model is described by the following equation:

(2) $$I_{s}([\theta \;T_{s}])=I_{c}(\theta_{0},T_{s})+\sum_{j=1}^{k}\theta_{j}\cdot I_{k}+e_{s}+\theta_{s}\cdot 1$$

where **θ** are the *k* + 1 linear combination coefficients for each pure spectrum and the bias coefficient to be estimated during the regression process. **I***_c_* is the continuous spectral component, modeled with Planck’s radiation law and physical and radiometric constants so that the radiation is in absolute units of µW/[nm∙cm^2^]. In this work, the model assumes that the continuous spectral component behaves as a gray body. Thus, a single parameter, *θ*_0_, represents the emissivity component, ε ≈ constant, as described by the following equation:

(3) $$I_{c}(\lambda ,[\theta_{0}\;T_{s}])=\theta_{0}\cdot \frac{c_{1}\cdot (\pi \cdot N.A.^{2})\cdot 10^{-7}}{\lambda^{5}\cdot \left[\exp\left(\frac{c_{2}}{\lambda \cdot T_{s}}\right)-1\right]}\left(\frac{\mu W}{{\mathrm{cm}}^{2}\mathrm{nm}}\right)$$

where *λ* is the wavelength in meters, *θ*_0_ is the relative gray body emissivity parameter and *T_s_* is the flame temperature in kelvin, to be estimated. The N.A. = 0.22 is the optical fiber numerical aperture and, *c*_1_ = 1.176∙10^−16^ (Wm^2^/sr) and *c*_2_ = 1.438786∙10^−2^ (m∙K) are the first and second Planck’s constants respectively. A scaling factor of 10^−7^ is included to ensure consistency of units with the measured spectra.

**Figure 3:**
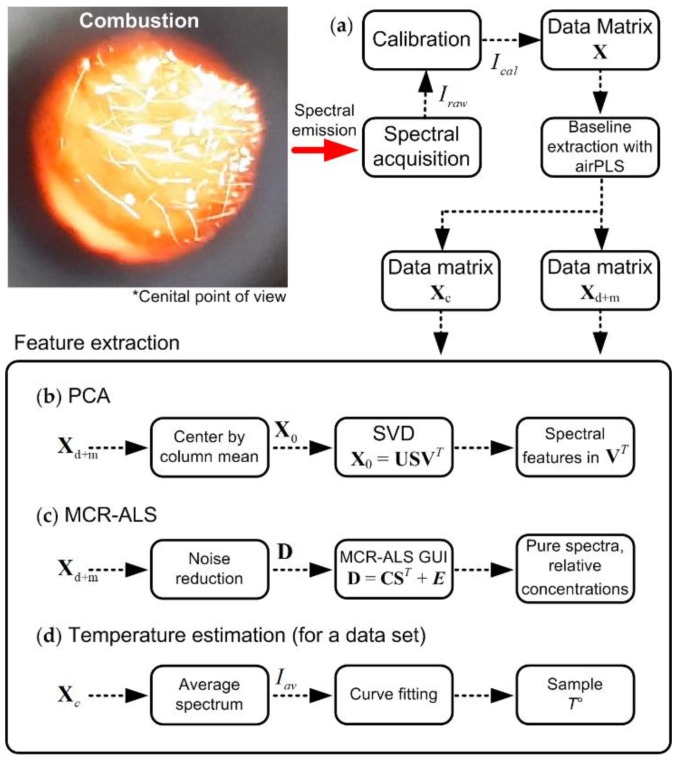
Data processing and analysis schemes implemented in this work: (**a**) Spectral signals acquisition and pre-processing, (**b**) PCA decomposition, (**c**) MCR-ALS implementation, (**d**) temperature estimation.

Although the aforementioned methods can be applied to the calibrated spectral data, pre-processing techniques were implemented to enhance specific spectral patterns. The airPLS (adaptative iteratively reweighted penalized least squares) baseline correction method [19] was implemented to separate the continuous spectrum from the discontinuous and band emissions. Other state-of-art algorithms were tested for such a purpose, however airPLS has shown the best results for the spectral features depicted in this work. Moreover, it was also verified that the convergence of optimization-based methods, e.g., MCR-ALS, was promoted by implementing noise reduction techniques in the data. In this work, the Savitzky-Goaly (SG) algorithm was implemented [20]. Figure 3 summarizes the different methodologies applied in this work.

## 3. Results and Discussion 

**Figure 4:**
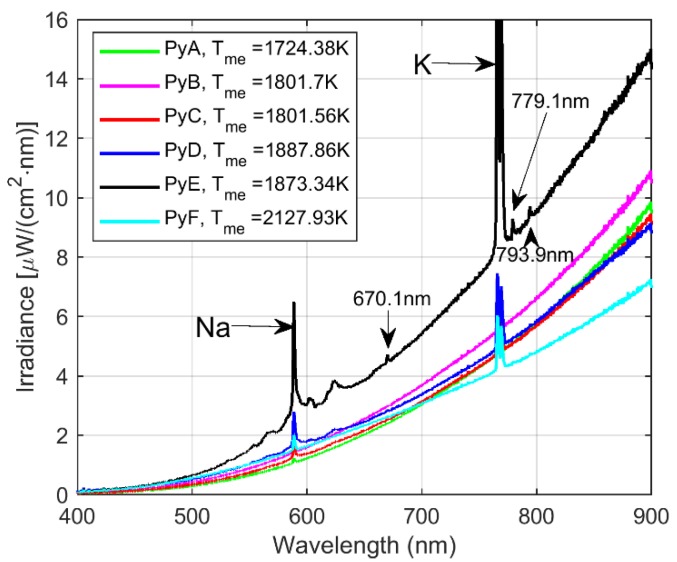
Average spectral emission from pyrite minerals in combustion experiments by considering different particle sizes.

Spectral signals from the foregoing experiments are analyzed in this section. The first step consisted on applying the airPLS baseline extraction algorithm to separately analyze both patterns. This algorithm was implemented with default parameter values, Matlab^TM^, and other platform implementations can be found in [21]. With the continuous features, a first approximation of the combustion temperature was obtained by fitting each spectrum in the spectral range to the model in (3). During the combustion experiments, irradiance intensity varied during the time scale. In this work, this issue is not addressed since the main goal was to detect spectral features to characterize the samples. In future work, the dynamical behavior of such patterns will be analyzed. Median temperature values for each test are reported in Figure 4. The average low-resolution spectra from the pyrite combustion experiments are displayed together with the corresponding median estimated temperature for each test. It is important to note that the spectral irradiance amplitude varied during the acquisition.

Note that some discontinuous sections with fundamentals and overtones can be seen in the average spectra, particularly, for particle sizes of PyD and PyE, it is possible to observe, molecular emission without the need of extra signal processing. Spectral line emissions corresponding to sodium (Na) around 589 nm and potassium (K) around 767 nm are normally reported in combustion experiment (considered as spectral interferences in this work and literature [22]). However, one of the hypotheses of the present work is the possibility of using these interferences as a trace for weak spectral features masked by noise and as a trace for the combustion flame presence in the FOV of the optical system. Furthermore, in the spectral range near potassium emission, only 2-line emissions appeared at 779.1 nm and 793.9 nm. The same is observed in Figure 4 for the PyE sample, from the U.S. Department of Commerce National Institute of Standards and Technology (NIST) Atomic Spectra Database website [23], which can be related to iron. Finally, the line emission at 670.1 nm can be also associated with Fe according to the NIST database.

In general, the estimated temperature of the combustion flame increased as the particle size fraction fed into the reactor was decreased. This could be explained in terms of the oxidation degree of the samples during the combustion given the exothermic nature of the oxidation reactions which were more extensive for the smaller particle size fractions, as reported in [18].

**Figure 5:**
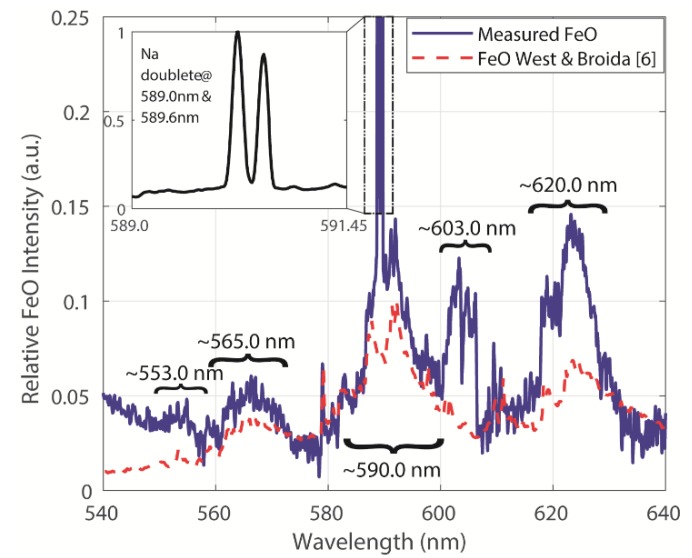
Spectral FeO emission measurements and reference spectral features.

Figure 5 depicts our high spectral resolution measurement of the possible FeO spectral emission. This signal clearly shows the sodium doublet profile at 589.0 nm and 589.6 nm, which was not recognizable in the lower spectral resolution measurements. Also, a laboratory measured FeO spectrum from West and Broida [7] is displayed together with our measurement. One can conclude that FeO emissions plus other chemical combustion components are recorded in our measurements. This figure also depicts a broad spectral emission around 603.0 nm, which will be analyzed later.

**Figure 6:**
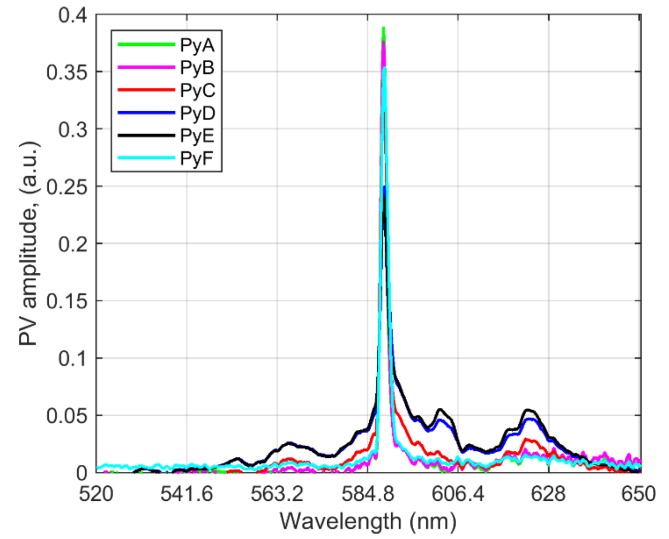
Extraction of spectral features with PCA from pyrite combustion for different particle sizes, P.V. 1 (loadings vectors) are depicted.

Analyzing the set of pyrite combustion experiments, an exploratory examination with PCA was applied to each baseline corrected-off measured data set. Therefore, it was possible to focus on the spectral features at Na and K fundamental emissions. In Figure 6, the results for the spectral range around the Na fundamental feature are shown. Note that large intensity variations are observed for different combusted particle sizes. Some spectral features are extremely enhanced, e.g., bands at ~553.0 nm, 565.0 nm, 590.0 nm and 620.0 nm. Some of these spectral features are related to FeO emission [7], as seen in Figure 5, and some others new appear, e.g., molecular bands ~ 603.0 nm.

**Figure 7:**
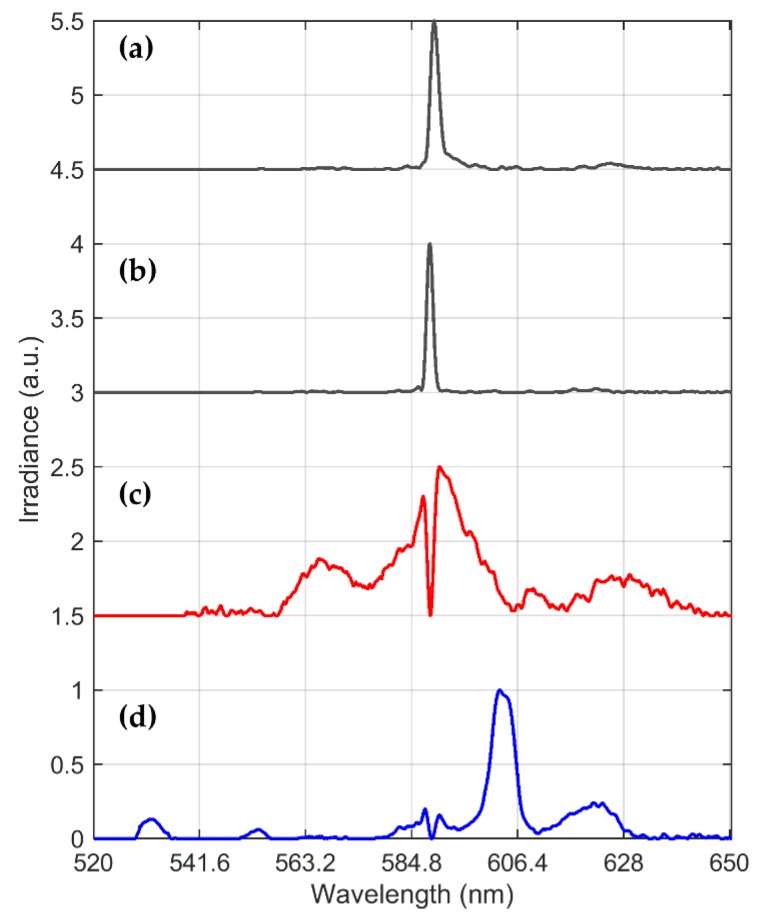
Extraction of spectral features with MCR-ALS from PyE pyrite sample combustion, each spectrum is normalized to its maximum: (**a**,**b**) are associated to a sodium doublet, named as **I**_1_ and **I**_2_ for subsequent analysis, (**c**) is associated with FeO emission, **I**_3_ and (**d**) emission of other iron oxides emission, **I**_4_. **Obs**. Spectral features are normalized to their maximum and offsets to each spectrum are included for comparison purposes.

Then, the MCR-ALS method was applied to the combustion experiment spectrum with the highest, in intensity, emission profile. In this case, spectra from the sample PyE were selected. The main settings selected for the MCR-ALS GUI were: the number of components to estimate, assessed with the SVD algorithm according to estimated eigenvalues, was 8. From an exploratory analysis, with an initial estimate of the concentration matrix with the “Pure” (purest variable detection method [24]) option, best spectral separation results were achieved. Non-negativity constraints were implemented over the spectra and concentration profiles. This was carried out with the fast-non-negative least squares algorithm (fnnls) [25]. With these settings, a 99.881% of the data variance and a fitting error (PCA) [15] of 1.2843% were achieved with 92 iterations. A total of 4 pure spectral components were selected as chemically meaningful based on their relative concentrations and their non overlapping spectral behavior, which are depicted in Figure 7. It can be seen that the FeO emission and sodium doublet are resolved from the decomposition and that an extra spectral feature, **I**_1_, is to be analyzed.

Now, the challenge is to correlate the spectral bands identified in the foregoing analysis to different iron oxides produced during pyrite combustion. To answer this question, let us note first that iron oxides such us wustite (FeO), magnetite (Fe_3_O_4_) and hematite (Fe_2_O_3_) contain different oxidation states of Fe: FeO, which contains only Fe(II) (nomenclature for Fe^2+^ ion), Fe_2_O_3_ contains only Fe(III) type of Fe and Fe_3_O_4_ contains both oxidation states, which is also referred as Fe(II,III). From a spectral standpoint, Table 2 summarizes the relevant emission bands and lines identified from NIST spectral line database and literature [23,26] that can be correlated to the signals found in Figure 7 signals. In particular, some of the emissions of Fe(III) are found in Figure 7d.

Secondly, the oxidation products collected in the receptacle located at the bottom of the reactor, were characterized in terms of their mineralogical composition by means of X-ray Diffraction (XRD) and QEMSCAN^®^. In all oxidized samples, the main mineralogical species identified were magnetite, hematite and in a smaller proportion, wustite and other substances. The mass contribution of Fe_3_O_4_ and Fe_2_O_3_ represented more than 92% of the overall weight in each sample.

As previously discussed, some of the emission signals shown in Figure 5, Figure 6 and Figure 7, were related to the FeO generation, while some others remained unknown. Based on the information of the composition of the oxidation products, the unknown signals may be associated to the formation of Fe_3_O_4_ and/or Fe_2_O_3_ during the ignition of the pyrite particle cloud.

The coexistence of magnetite and hematite in the products could be explained from the analysis of the well-known phase diagram of the Fe-O system [27]. For the average estimated temperatures of the combustion flames (>1724 K) and the samples oxygen content (28.5 to 30 pct. by weight), an ionic and homogeneous liquid composed of Fe(II), Fe(III) and oxygen, was formed as a result of the particle cloud ignition. As the molten particles were cooled, the precipitation of magnetite, as the first solid iron oxide, had occurred. Then, as the particles temperature of the particles continued to decrease (until they reached the receptacle), the formation of two solid phases, occurred. The solid phases were magnetite and hematite, and they represent the state of the samples at the moment of their collection and further characterization.

**Figure 8:**
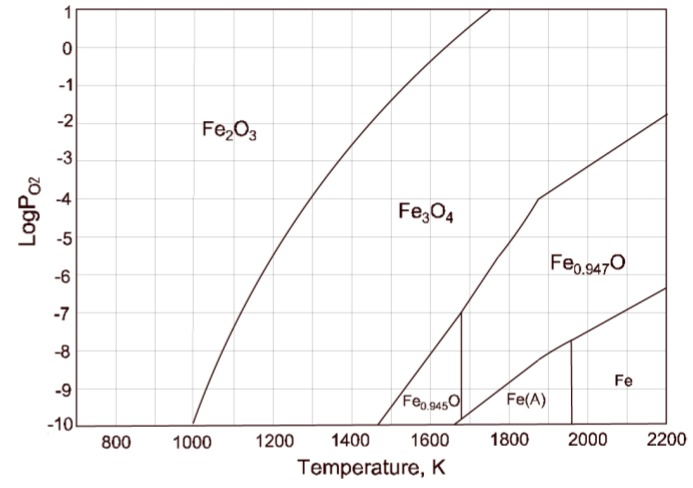
Predominance diagram for the Fe-O system at high temperatures constructed with HSC^®^ software. The sections delimited by the continuous lines, represent the conditions at which the described species are thermodynamically stable.

Additionally, in Figure 8, a predominance diagram for the Fe-O system, is presented. According to this diagram, at high temperatures (>1700 K), under the highly oxidant conditions (oxygen potential, log P_O2_, close to zero), the only stable species in the combustion flame were magnetite (Fe_3_O_4_) and wustite (indicated in Figure 8 as Fe_0.947_O and Fe_0.945_O). Therefore, the formation of hematite (Fe_2_O_3_) must have occurred during the cooling stage of the oxidation products, since this iron oxide can exist only at low temperatures (<1500 K).

**Figure 9:**
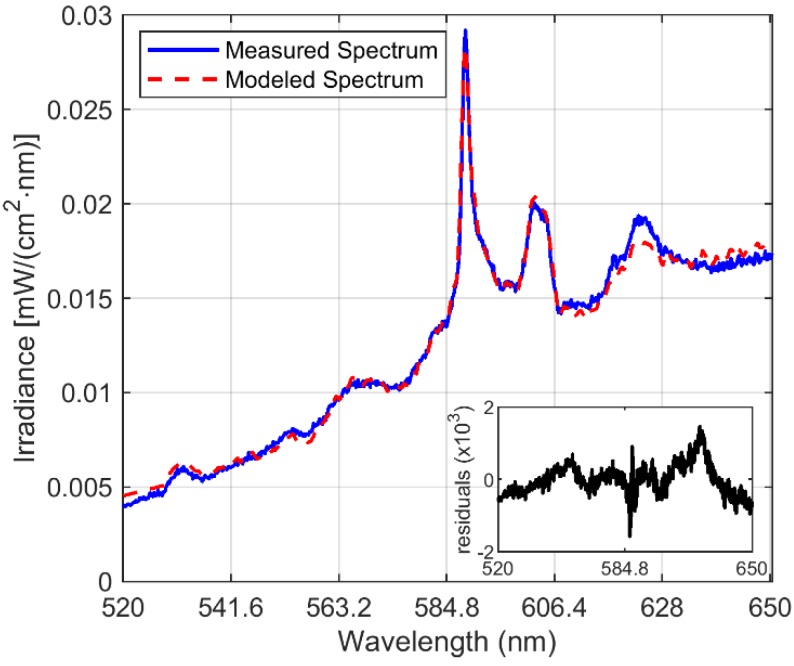
Non-linear curve fitting results for a spectrum from combustion of PyE sample, RMSE (root mean square error) = 0.4393, *θ*_0_ = 0.0249, *T_s_* = 220.3 K, *θ*_1_ (related to **I**_1_ or Na) = 3.4583, *θ*_2_ (related to **I**_2_ or Na) = 15.7373, *θ*_3_ (related to **I**_3_ or FeO) = 6.2484, *θ*_4_ (related to **I**_4_ or Fe_3_O_4_) = 7.3093 and *θ*_5_ (related to an offset) = 9.6 × 10^−8^.

Thus, we claim that the foregoing oxidation process discussion, provides sufficient thermodynamic analysis to relate the unknown spectral emission signal in Figure 7d to the formation of magnetite. On the other hand, the aforementioned results can be employed to initially address the issue of on-line copper combustion process monitoring. In order to do this, the model proposed in Equation (2) can be applied to generate, for example, an alert that the combustion is running in an overoxidation state. Here, to estimate the proposed model parameters, including the four estimated pure components depicted in Figure 7, a non-linear fitting procedure such as ${\left\|I_{s}(\lambda ,[\theta \;T_{s}])-I_{m}\right\|}_{2}^{2}$ is minimized. The selected optimization algorithm was the trust-region-reflective, which can be implemented with built-in Matlab^TM^ optimization toolbox functions. Positive constraints were included, since the relative concentrations, the gray body emissivity, the flame temperature and the signal offset are all positive for each spectrum. The non-zero selected initial conditions were an emissivity of 0.5, and a temperature of 1500 K. A fitting result for the sample PyE combustion spectrum can be seen in Figure 9. Note that the model accounts for most of the signal structure. As depicted in the residual plot (down-left of inside Figure 9), there is still information non modeled by (2) and (3) that can be explained by errors introduced by the gray body assumption, and by the lack of fitting from the signal separation procedure. Furthermore, note that by following the proposed method, the model in (2) can be extended to include other spectral features in the analyzed spectral range, such as the emission of Cu_x_O molecules that are expected to appear in practical industrial flames. The foregoing will be investigated in future work.

**Figure 10:**
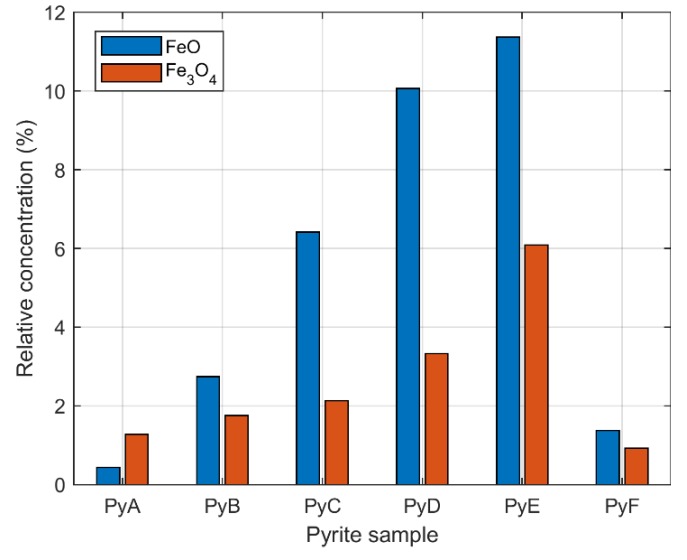
Relative concentrations (median values for each data set) of FeO and Fe_3_O_4_ band emissions estimated from spectra of pyrite combustion. Obs. Sample particle sizes decrease from left to right.

Then, the regression model was applied to the other data sets and the results for FeO and Fe_3_O_4_ are summarized in Figure 10, where the relative concentrations of the four pure spectrum depicted in Figure 7 are normalized as *θ*_j_ = *θ*_j_/Σ(*θ*_j_), j = 1:4, and expressed as a percentage for each spectral sample. Median values are displayed since the experimental results presented skewed distributions.

From Figure 10, the relative concentrations for the two molecular emissions are highly correlated, with a correlation coefficient of 0.9134, no correlation was found with the sodium doublet or estimated temperature. A nonlinear relation between the estimated concentrations and particle size was observed, as well as the estimated temperature. On the other hand, by following the aforementioned procedures, drop-tube wall spectral emission was analyzed. It was verified that the background radiation is only characterized by a continuous spectral component. Thus, spurious radiation that could affect the molecular or the emission lines from the process are discarded. Moreover, since the emitted radiation at the reported experimental temperatures reaches its maximum in the infrared spectral range (e.g., by Wien’s Displacement law, the radiation from a black body at 1800 K reaches its spectral peak at ≈1.6 µm), it could contain important process information that was not depicted in the visible region. This hypothesis will be explored in future work.

Finally, it should be noted that the practical application of the proposed approach is aimed to develop tools to monitor and characterize the oxidation state of copper concentrates during their combustion in flash furnaces, allowing operators to have online feedback from the process. Furthermore, note that the reported approach, summarized as: (i) the acquisition of spectral signals; (ii) the pre-processing and calibration of such signals; (iii) the extraction of meaningful features/patterns from measured spectra with a multivariate curve resolution method and; (iv) the application of a non-linear regression method with the models in (2) and (3) to estimate the combustion temperature, relative emissivity and relative concentrations of spectral patterns contained in each measured spectrum, can be extrapolated to other combustion processes, with the constraint that different fuels will generate different spectral features during their combustion, so an understanding about the involved chemical reactions and physical transformations is needed.

## 4. Conclusions

In this work, an experimental study aimed at identifying and characterizing the spectral features emitted during the combustion of iron sulfide pyrite was performed. Spectral signals and bands were related to the formation of wustite and magnetite based on the composition of the reacted products, a comparison with spectra reported in the literature, and a thermodynamic discussion that provided information of the oxidation process. Moreover, a high spectral resolution spectrometer was developed to resolve the Na emission doublet, and to validate a technique based on spectra acquired by the low-resolution spectrometer plus signal processing methods. With the resolved pure spectral signals by means of the MCR-ALS method and a proposed model describing the spectral emission of these minerals during combustion, relative concentrations of spectral features and combustion temperature were estimated with a non-linear least square fitting procedure for each acquired spectrum. The proposed methodology demonstrated to be effective for signal reconstruction and, in future work, it will be applied for the analysis of industrial spectral data. The proposed model will be refined to include spectral emissivity assumptions based on physical laws. Furthermore, we conclude that the proposed techniques are a feasible alternative to monitor and to control the flash smelting process and to characterize materials under oxidation conditions, particularly, the ones containing pyrite, such as copper concentrates. Finally, we claim that this is the first reported Fe_3_O_4_ visible spectral emission measurement achieved during its formation in iron sulfide pyrite combustion.

## Figures and Tables

**Table 1:** Developed spectrometer main characteristics.

Characteristic	Value
Spectral range 1: VIS	[532.99–640.48] nm
Spectral range 2: NIR	[727.45–827.07] nm
Spectral resolution (Full Width at Half Maximum, FWHM)	0.12 nm
Sampling density	0.03 nm/pixel
Diffraction grating	1200 l/mm @500 nm, holographic
Detector	Toshiba TCD1304AP
Number of pixels	3648
Pixel size	8 × 200 µm (29.184 × 0.2 mm)
Slit width	20 µm × 5 µm
Operating temperature	20 °C

**Table 2:** Spectral emission lines and bands emission identification (*).

Molecule/Element or Ion	Emission Center Wavelength, nm
Fe I	565.9, 602.4, 619.2
Fe II	531.6, 566.0, 623.8
Fe III	531.1, 535.4, 583.4, 600.0, 603.3
FeO [6,7,8,9,21]	564.7, 579.0, 586.8, 590.3, 597.5, 609.7, 611.0, 618.0, 621.8

* Emission bands for magnetite and hematite at the reported temperatures were not found in the literature.
