# Erratum: Toro, C. et al. On the Detection of Spectral Emissions of Iron Oxides in Combustion Experiments of Pyrite Concentrates. *Sensors* 2020, *20*, 1284

**DOI:** 10.3390/s20216141

**Published:** 2020-10-28

**Authors:** Carlos Toro, Sergio Torres, Víctor Parra, Rodrigo Fuentes, Rosario Castillo, Walter Díaz, Gonzalo Reyes, Eduardo Balladares, Roberto Parra

**Affiliations:** 1 Dirección de Investigación, Universidad Tecnológica de Chile INACAP, Avenida el Condor 720, Ciudad Empresarial, Huechuraba, Santiago RM858000, Chile; 2 Metallurgical Engineering Department, University of Concepción, Concepción CCP4070386, Chile; vparras@udec.cl (V.P.); walterdiaz@udec.cl (W.D.); gonzaloreyes@udec.cl (G.R.); eballada@udec.cl (E.B.); rparra@udec.cl (R.P.); 3 Electrical Engineering Department, University of Concepción, Concepción CCP4070386, Chile; sertorre@udec.cl; 4 Department of Analytical and Inorganic Chemistry, Faculty of Chemical Sciences, University of Concepción, Concepción CCP4070386, Chile; rodrigoal@udec.cl; 5 Department of Instrumental Analysis, Faculty of Pharmacy & Biotechnology Center, University of Concepción, Concepción CCP4070386, Chile; rosariocastillo@udec.cl

The authors wish to make the following corrections to this paper [1]: we have found a typing error in the caption of Figure 9 (page 12)—*T*_s_ should have a value of 2220.3 K, not 220.3 K.

The correct caption of Figure 9 is

**Figure 9.** Non-linear curve fitting results for a spectrum from combustion of PyE sample, RMSE (root mean square error) = 0.4393, *θ*_0_ = 0.0249, *T*_s_ = 2220.3 K, *θ*_1_ (related to **I**_1_ or Na) = 3.4583, *θ*_2_ (related to **I**_2_ or Na) = 15.7373, *θ*_3_ (related to **I**_3_ or FeO) = 6.2484, *θ*_4_ (related to **I**_4_ or Fe_3_O_4_) = 7.3093 and *θ*_5_ (related to an offset) = 9.6 × 10^-8^.

This change has no material impact on the conclusions of our paper. The authors would like to apologize for any inconvenience caused to the readers by this change.
